# Behavioral impact of condensed repeated ethanol dosing on withdrawal in adult zebrafish (*Danio rerio*)

**DOI:** 10.3389/fnsys.2026.1924496

**Published:** 2026-09-11

**Authors:** Rawlie Prince, Tobin Steman, Ethan V. Hagen, Andréa L. Johnson, Yanbo Zhang, Trevor J. Hamilton

**Affiliations:** 1 Department of Psychology, MacEwan University, Edmonton, AB, Canada; 2 Department of Psychiatry, University of Alberta, Edmonton, AB, Canada; 3 Neuroscience and Mental Health Institute, University of Alberta, Edmonton, AB, Canada; 4 Allen Discovery Center for Neurobiology in Changing Environments, University of California, San Diego, CA, United States

**Keywords:** addiction, anxiety-like behavior, aquatics, behavioral neuroscience, boldness, locomotion, pharmacology

## Abstract

Zebrafish (*Danio rerio*) are a widely used animal model to study a variety of neuropsychiatric disorders, including drug addiction. Existing paradigms to examine alcohol addiction use repeated daily or continuous ethanol exposures over multiple days. The present study evaluated a condensed repeated dosing paradigm, involving three 1-h ethanol doses (0.8%) per day over 5 days. To examine ethanol withdrawal, locomotor and anxiety-like behavior was assessed at 2, 4, and 8 days following the dosing schedule, using the open field test. There was a significant increase in immobility and angular velocity with ethanol regardless of withdrawal duration. At 8 days of withdrawal, ethanol-treated fish spent significantly less time highly mobile compared to controls. Likewise, there was a change in zone preference at 8 days of withdrawal, with ethanol-treated fish spending significantly more time in the transition zone and less time in the thigmotaxis zone compared to controls. No other significant zone preferences or sex differences were found. These findings suggest that the condensed repeated dosing paradigm produces a measurable behavioral withdrawal phenotype. This dosing schedule is an effective and efficient approach for high-throughput drug addiction research in adult zebrafish and may be useful for evaluating many pharmacological treatments.

## Introduction

Human consumption of alcohol dates to at least 7000 BC, making it the most widely consumed, legally accessible, and culturally ingrained psychoactive recreational drug (Hendriks, 2020; McGovern et al., 2004). Ethyl alcohol (ethanol) is a psychoactive substance whose primary effect is depression of the central nervous system through the activation, inhibition, and modulation of multiple types of pre- and post-synaptic receptors (Vengeliene et al., 2008). Both acute and chronic alcohol use are associated with negative health outcomes and impose a significant economic and social burden on society through increased healthcare costs, loss of productivity, disability, and premature mortality (Baumberg, 2006; Stahre, 2014). Alcohol Use Disorder (AUD) is a condition defined by the DSM-5-TR as a pattern of problematic alcohol use leading to clinically significant impairment or distress (American Psychiatric Association, 2022). Cessation of prolonged alcohol use often results in withdrawal, which is critical to manage in the treatment of AUD (Carvalho et al., 2019; Jesse et al., 2016). Depending on the severity of alcohol dependence and the degree of subsequent neuroadaptation, withdrawal can range from mildly uncomfortable to life-threatening (Chapman and Plaat, 2009; Jesse et al., 2016). Therefore, AUD withdrawal management requires close clinical monitoring and medical treatment (Wood et al., 2023). Although multiple pharmacological treatments for AUD and withdrawal exist, their undesirable side effects and long treatment durations continue to prompt the development and testing of new therapeutic approaches (Swift and Aston, 2015; Jesse et al., 2016; Witkiewitz et al., 2019).

Animal models and their associated paradigms have substantial utility in the drug discovery and refinement process (Nainu et al., 2023). Repeated cycles of ethanol administration and cessation have been commonly used in animal models to evoke withdrawal effects, which often lead to anxiety-like behavior (Clayman et al., 2017; da Silva Chaves et al., 2018; Hagen et al., 2025; Müller et al., 2017; Nowicki et al., 2015; Tran and Gerlai, 2013). Given the complexity surrounding the specific drinking patterns and conditions that lead to AUD and subsequent withdrawal symptoms, there is likewise ambiguity in the organisms and associated paradigms designed to simulate the disorder (da Silva Chaves et al., 2018; Knapp and Breese, 2012). Classical mammalian models using rats or mice as subjects vary significantly, with some utilizing *ad libitum* paradigms (Fachin-Scheit et al., 2006; Knapp and Breese, 2012), gaseous ethanol administration (Becker and Lopez, 2004), ethanol-supplemented diets (Wills et al., 2009), or intravenous ethanol injections (Fukushiro et al., 2012) all with their strengths and weaknesses.

Zebrafish (*Danio rerio*) are well established as a powerful and effective model organism; their high fecundity, low maintenance costs, fully sequenced genome with approximately 70% protein-coding gene homology with humans, and ability to absorb water-soluble chemicals make them an increasingly popular choice for neuropharmacological research (Bauer et al., 2021; Hughes and Hessel, 2024; Johnson et al., 2023, 2025; Howe et al., 2013; Johnson and Hamilton, 2017). The ease and consistency with which zebrafish can be dosed with ethanol also make them an ideal high-throughput model for studying AUD (Krook et al., 2019). Acute ethanol exposure in zebrafish has demonstrated anxiolytic-like behavioral changes, whereas chronic ethanol exposure and subsequent withdrawal have been associated with anxiogenic-like behavioral changes (Cachat et al., 2010; Holcombe et al., 2013; Krook et al., 2019; Mathur and Guo, 2011; Müller et al., 2017; Pittman and Ichikawa, 2013; Tran et al., 2015). The open field (OF) test is well-validated and widely used to assess anxiety-like behavior in zebrafish (Blaser et al., 2010; Godwin et al., 2012; Johnson and Hamilton, 2017; Stewart et al., 2012).

A limitation of current AUD withdrawal paradigms in zebrafish is their reliance on repeated ethanol exposure over relatively long durations. Many studies involve a repeated daily exposure or continuous (chronic) exposure for 1–3 weeks (Table 1). The goal of the current study was to develop and evaluate a condensed ethanol dosing paradigm with three 1-h administrations per day over 5 days to shorten the dosing period and strengthen translational relevance by more closely aligning with AUD drinking patterns in humans (NIAAA, 2001). Locomotion and anxiety-like behavior were assessed using the OF test after either 2, 4, or 8 days of withdrawal.

**TABLE 1:** Summary of relevant factors from zebrafish chronic alcohol exposure paradigms in literature.

Study	Assay	Dosing schedule	Ethanol concentration (vol/vol)	Paradigm duration	Withdrawal duration
Dlugos and Rabin (2003)	NNT	Chronic	0.5%	14 days	N/A
Gerlai et al. (2009)	NTT, group preference, aggression, predator test	Chronic	0.125%, 0.250%, 0.375%, and 0.50%	22 days	1 h
Cachat et al. (2010)	NTT	Chronic	0.3%	7 days	3 h per day for 7 days
Dlugos et al. (2011)	NNT	Chronic	0.5%	70 days	N/A
Mathur and Guo (2011)	NTT, LDT	20 min daily	1.00%	8 days	24–168 h
Holcombe et al. (2013)	LDT	30 min daily or weekly	0.2% (daily) 1.4% (weekly)	21 days	48 h
Pittman and Ichikawa (2013)	NTT, LDT	60 min daily	3.00%	14 days	48 h
Tran and Gerlai (2013)	NTT	Chronic	0.125%, 0.250%, 0.375%, and 0.50%	22 days	1 h
Pittman and Hylton (2015)	NTT	60 min daily	3.0%	14 days	48 h
Dewari et al. (2016)	NTT	Chronic	0.5%	63 days	1512 h
Clayman et al. (2017)	NTT, LDT, shoaling test	20 min daily	1.00%, 1.25%, 1.5%, 1.6%, or 1.75%	7 days	N/A
Müller et al. (2017)	Mitochondrial O_2_ consumption	20 min daily	1.00%	8 days	N/A
da Silva Chaves et al. (2018)	LDT, catalase activity	Chronic	0.125%–0.5% (doubled every 4 days)	16 days	1 h
Krook et al. (2019)	NOT	60 min daily	0.4% or 0.8%	21 days	48 h
Kitson et al. (2022)	NTT, OFT	30 min daily	1.00%	7 days	N/A
Hagen et al. (2025).	Conditioned color preference	60 min daily	0.8%	21 days	48–192 h
Resmim et al. (2025)	OFT	20 min daily	1%	7 days	24 h

NTT, novel tank test; NNT, nearest neighbor test; LDT, light/dark test; OFT, open field test; NOT, novel object approach test.

## Materials and methods

This experiment used a 2 × 3 × 2 factorial design with three independent variables: ethanol treatment (0.8% (vol/vol) ethanol or habitat water control), days of withdrawal (DW) when tested (2, 4, or 8), and sex (male or female). The experiment was carried out in two distinct experimental sets, 1 month apart. Each set consisted of three ethanol experimental groups with varying withdrawal durations (*n* = 12 per group) and their appropriate negative controls (*n* = 12 per group).

### Subjects

Adult zebrafish, AB/wildtype (*Danio rerio*; *n* = 144) aged 6–7 months, were bred in-house in the MacEwan fish facility. All fish were housed in one of four interconnected Techniplast ZebTEC multilinking systems (Techniplast Group, Toronto, ON, Canada) racks in 3 L or 10 L tanks. All tanks were connected to a central water treatment unit (Techniplast) that circulated, aerated, and automatically changed the water. Habitat water pH was set to 7.1 and ranged from 6.7 to 7.1; temperature was set to 28.5 °C and maintained between 27 °C and 29 °C. The day/night cycle was set to a 14-h light cycle with lights on at 7:00 and off at 21:00. Zebrafish were fed fish pellets (Gemma Micro 300, Skretting by Nutreco, Westbrook, USA) twice daily (∼09:00 and ∼15:00), except during the dosing period, when fish were fed only once per day after the final dose (∼15:00). Fish were sexed based on coloration and body morphology (Spence et al., 2008). Sexing was completed during sample selection and confirmed prior to behavioral testing and again during dissection. Zebrafish were randomly selected from 10 L housing tanks and placed into 3 L tanks in groups of 12 (∼50:50 males and females). There were two replicate tanks per condition, for a total of 12 tanks, which was performed in two identical experimental sets. Sample sizes (*n* = 24 per condition) were based on previous research (Holcombe et al., 2013; Tran and Gerlai, 2013; Krook et al., 2019). Each set of fish was housed in their respective tank in the aquatic habitat on the same tier for the duration of the experiment. All fish were naïve to experimental testing and pharmacological intervention. All experiments were approved by the MacEwan University Animal Research Ethics Board (AREB) under protocol number 05-12-13 in compliance with the Canadian Council for Animal Care (CCAC) guidelines for the care and use of experimental animals.

### Condensed repeated ethanol dosing

**FIGURE 1:**
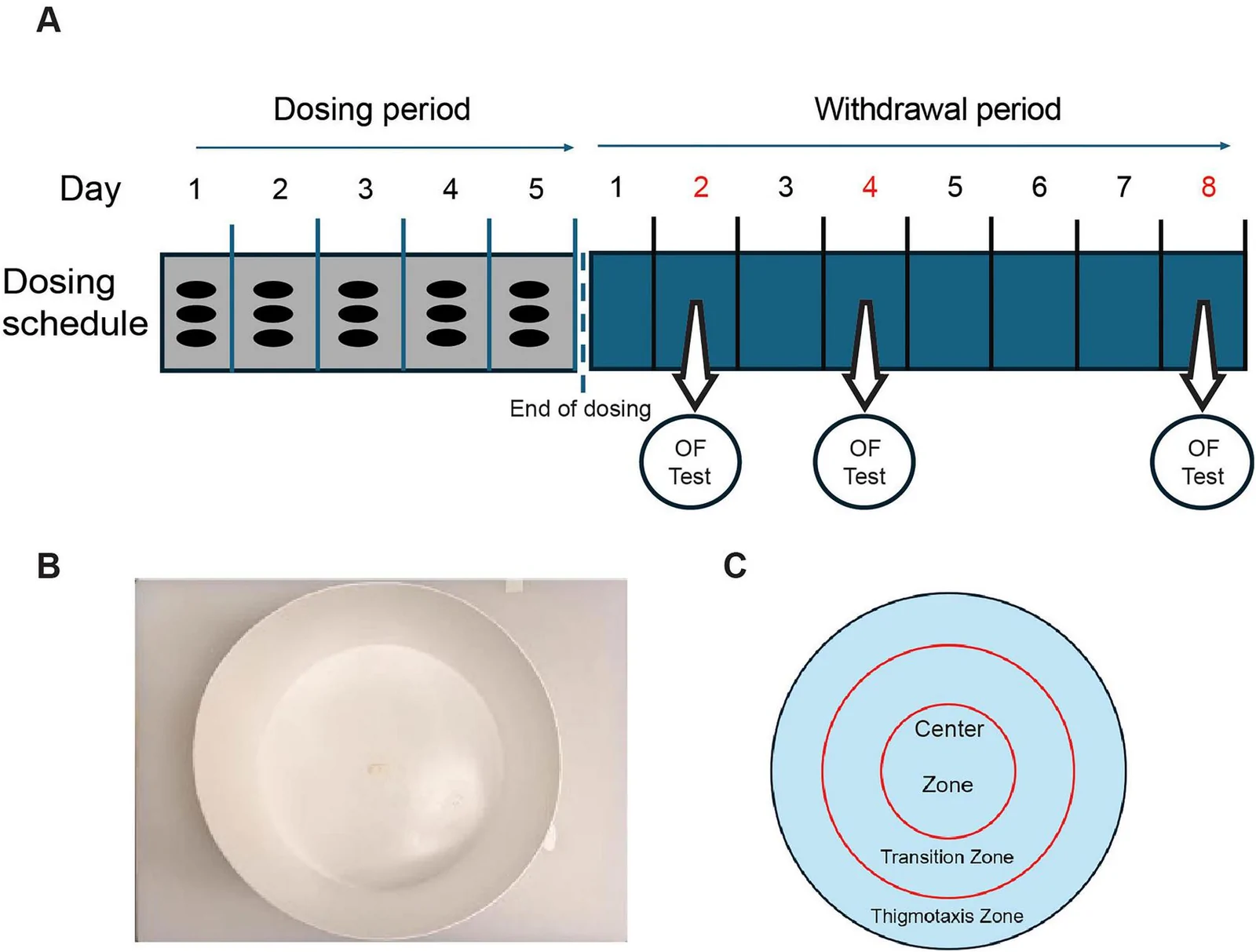
Ethanol dosing and testing apparatus. **(A)** Experimental timeline. Adult zebrafish were dosed for 1 h three times daily for 5 days with either 0.8% (vol/vol) ethanol or habitat water. Following the final dose, separate groups of fish underwent behavioral testing after 2, 4, or 8 days of withdrawal. **(B)** Open field test arena used for behavioral assessment (diameter 26.3 cm, wall height: 15 cm). **(C)** Diagram of the open field arena and the zones used in the analysis.

Ethanol-exposed and habitat water control fish were dosed three times daily (09:00, 12:00, and 15:00) over five consecutive days (Figure 1A). Ethanol exposure was set to 0.8% (vol/vol) based on previous studies withdrawal in zebrafish (Krook et al., 2019; Hagen et al., 2025). Following the final dose, fish were assigned to withdrawal periods of 2, 4, or 8 days prior to behavioral testing (Figure 1A). A perforated spawning insert was placed into each of the six housing tanks to permit dosing according to the method outlined by Holcombe et al. (2013). Experimental dosing tanks were prepared by adding appropriate volumes of 95% (vol/vol) ethanol directly to tanks containing habitat water up to 1 L. In contrast, control dosing tanks consisted solely of 1 L of habitat water. All groups, including controls, were transferred from habitat tanks to dosing tanks via spawning inserts and left for 1 h (Hagen et al., 2025). Upon completion of the dosing period, each group was transferred to a tank of habitat water for a 5-min rinse prior to being returned to their home tanks. Heating pads (Hydrofarm, Petaluma, USA) were placed under the tanks to maintain water temperature at 26 °C –28 °C during dosing periods. Additionally, a dosing area was created using white corrugated plastic barriers to minimize any external visual stimuli. No mortality occurred over the duration of the experiment.

### Behavioral testing

The OF test was used to measure locomotion and anxiety-like behavior in individual zebrafish. The testing arena consisted of a white plastic cylinder (26.3 cm diameter, 15 cm walls) with a habitat water depth of 5 cm, based on previous studies (Figures 1B, C; Johnson and Hamilton, 2017; Krook et al., 2019). Behavioral testing was conducted 2, 4, or 8 days following the dosing period. On each day of testing, the appropriate 3L tanks were moved to a recording room adjacent to the habitat room, placed on a heating pad (Hydrofarm, Petaluma, USA) to maintain water temperature, with at least 20 min of acclimation prior to testing. Researchers were not blind to the treatment groups during dosing or testing. White corrugated plastic was placed around the tanks to mitigate potential confounding visual stimuli. After acclimation, fish were individually netted and gently placed into the testing arena with no more than 2 s of transfer time. Recordings began immediately and trials lasted 10 min using the differencing method in EthoVision XT (version 16; Noldus, Leesburg, VA, USA) motion tracking software. The dependent variables measured during the test were: time spent in the center (0–7.9 cm in diameter), transition (7.8–16.3 cm in diameter), and thigmotaxis (near the wall; 16.4–26.3 cm in diameter) zones (s), as well as distance travelled (cm), mean absolute angular velocity (deg/s), high mobility (s) and immobility (s), defined using frame-to-frame changes in fish pixels with thresholds of 60% for high mobility and 5% for immobility (Krook et al., 2019; Hagen et al., 2024). Following each trial, fish were placed into 600 mL beakers containing habitat water and sex was determined.

### Statistical analysis

Statistical significance was set at α = 0.05 with a 95% confidence interval. All data sets were assessed for normality using a D’Agostino and Pearson omnibus normality test. To examine differences between control and experimental groups, a mixed-effects analysis was used to assess the effects of treatment, withdrawal duration, and sex on behavior for all variables, with the Holm-Šídák procedure used for multiple-comparison *post hoc* tests. *Post hoc* analyses were conducted only where significant interaction effects were observed (main effects were not followed up with only two levels). Comparisons not relevant to the interaction of interest were not tested. Nested one-way ANOVA tests with Tukey’s multiple-comparison *post hoc* tests were used to verify homogeneity of replication rounds prior to pooling data. Data was analyzed with GraphPad Prism software (Version 10; GraphPad, San Diego, CA, USA) and is included in the Supplementary material. There were no significant differences between replicate groups, so data was pooled.

## Results

This study exposed zebrafish to ethanol (0.8% vol/vol) or habitat water (controls) for a 1-h period three times daily for 5 days. Separate groups of fish underwent an OF test at 2, 4, or 8 days of ethanol withdrawal. Behavioral variables measured were time spent in the center, transition, and thigmotaxis zones, the time spent in highly mobile and immobile states, mean absolute angular velocity, and total distance travelled.

### OF test: time in zones

#### Time in the center zone

**FIGURE 2:**
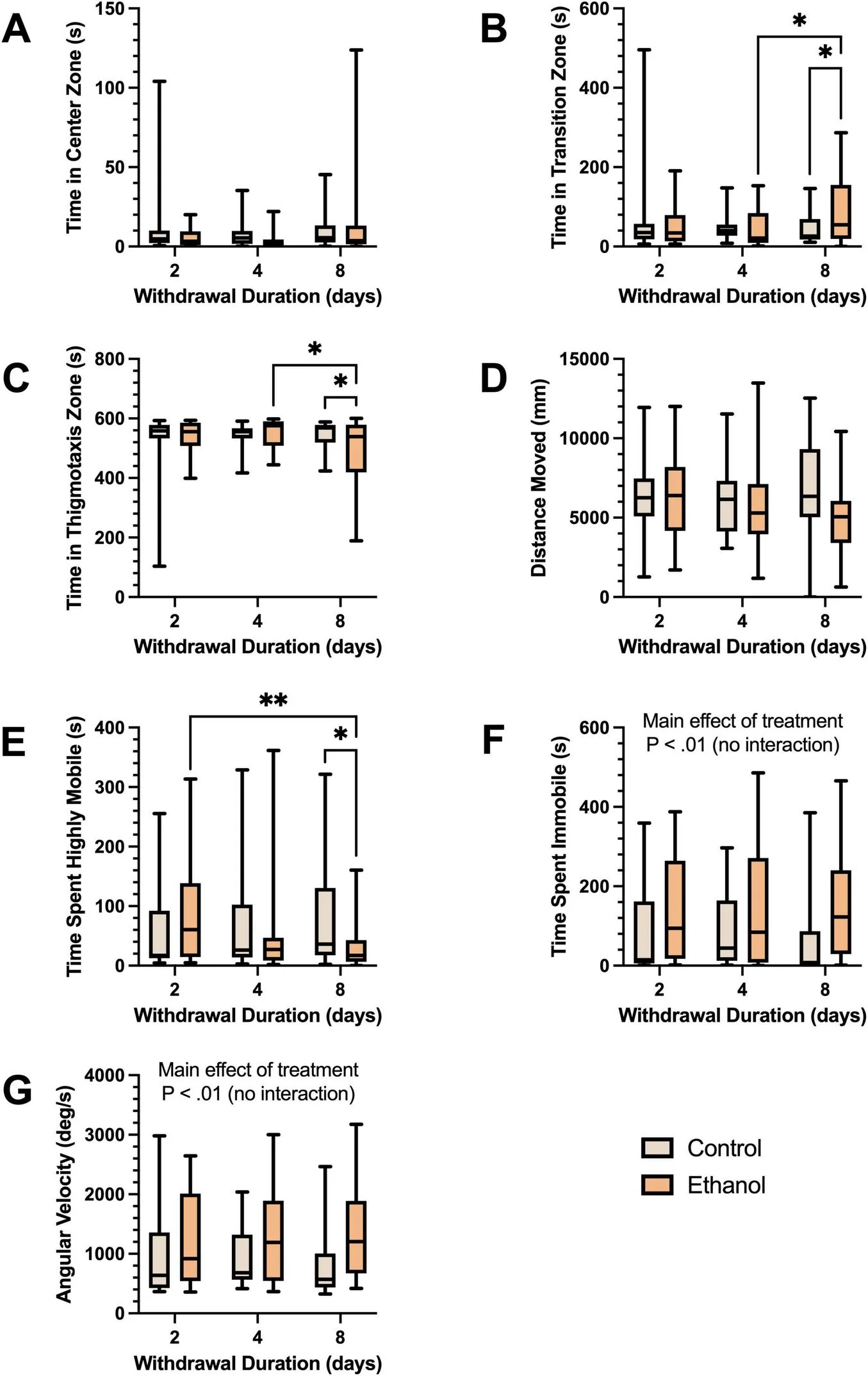
Condensed repeated ethanol dosing effects in the open field test. Fish were assessed at 2, 4, and 8 days of withdrawal following repeated dosing. **(A)** Time spent in the center zone of the arena. **(B)** Time spent in the transition zone of the arena. **(C)** Time spent in the thigmotaxis zone of the arena. **(D)** Total distance moved during the trial. **(E)** Time spent highly mobile. **(F)** Time spent immobile. **(G)** Mean absolute angular velocity. Each box represents the 25^th^ and 75^th^ percentiles, with whiskers showing the smallest and largest values. The median is represented by the line within each box. Asterisk indicate a significant difference (*P < 0.05, **P < 0.01).

A two-way mixed-effects analysis revealed no significant withdrawal duration × treatment interaction effect (*F*(2, 92) = 1.41, *p* = 0.250), and no significant main effects for withdrawal duration (*F*(2, 92) = 2.49, *p* = 0.088) or treatment (*F*(1, 46) = 0.15, *p* = 0.700; Figure 2A).

#### Time in the transition zone

A two-way mixed-effects analysis revealed a significant withdrawal duration × treatment interaction effect (*F*(2, 92) = 3.40, *p* = 0.038), but no significant main effects for withdrawal duration (*F*(2, 92) = 1.52, *p* = 0.225) or treatment (*F*(1, 46) = 0.38, *p* = 0.543; Figure 2B). A Holm-Šídák *post hoc* test revealed significantly increased time spent in the transition zone in ethanol-treated fish compared to control fish at 8 days of withdrawal (*p* = 0.027) and compared to ethanol-treated fish at 4 days of withdrawal (*p* = 0.034).

#### Time in the thigmotaxis zone

A two-way mixed-effects analysis revealed a significant withdrawal duration × treatment interaction effect (*F*(2, 92) = 3.72, *p* = 0.028), but no significant main effects for withdrawal duration (*F*(2, 92) = 2.09, *p* = 0.130) or treatment (*F*(1, 46) = 0.24, *p* = 0.624; Figure 2C). A Holm-Šídák *post hoc* test revealed significantly decreased time spent in the thigmotaxis zone in ethanol-treated fish compared to control fish at 8 days of withdrawal (*p* = 0.025) and compared to ethanol-treated fish at 4 days of withdrawal (*p* = 0.014).

### OF test: locomotion

#### Distance moved

A two-way mixed-effects analysis revealed no significant withdrawal duration × treatment interaction effect (*F*(2, 92) = 1.28, *p* = 0.284), or main effects of withdrawal duration (*F*(2, 92) = 0.58, *p* = 0.565) or treatment (*F*(1, 46) = 3.07, *p* = 0.086; Figure 2D).

#### Time spent highly mobile

A two-way mixed-effects analysis revealed a significant withdrawal duration × treatment interaction effect (*F*(2, 92) = 5.30, *p* = 0.007), but no significant main effects for withdrawal duration (*F*(2, 92) = 0.97, *p* = 0.384) or treatment (*F*(1, 46) = 0.17, *p* = 0.684; Figure 2E). A Holm-Šídák *post hoc* test revealed significantly decreased time spent highly mobile in ethanol-treated fish compared to control fish at 8 days of withdrawal (*p* = 0.039) and compared to ethanol-treated fish at 2 days of withdrawal (*p* = 0.005).

#### Time spent immobile

A two-way mixed-effects analysis revealed no significant withdrawal duration × treatment interaction effect (*F*(2, 92) = 0.52, *p* = 0.596) or main effect of withdrawal duration (*F*(2, 92) = 0.02, *p* = 0.978); however, a significant main effect of treatment was revealed (*F*(1, 46) = 7.56, *p* = 0.009; Figure 2F).

#### Mean absolute angular velocity

A two-way mixed-effects analysis revealed no significant withdrawal duration × treatment interaction effect (*F*(2, 92) = 0.67, *p* = 0.514), or main effect of withdrawal duration (*F*(2, 92) = 0.16, *p* = 0.854). However, a significant main effect of treatment (*F*(1, 46) = 7.52, *p* = 0.009; Figure 2G) was revealed.

### OF test: sex differences

#### Time in the center zone

**FIGURE 3:**
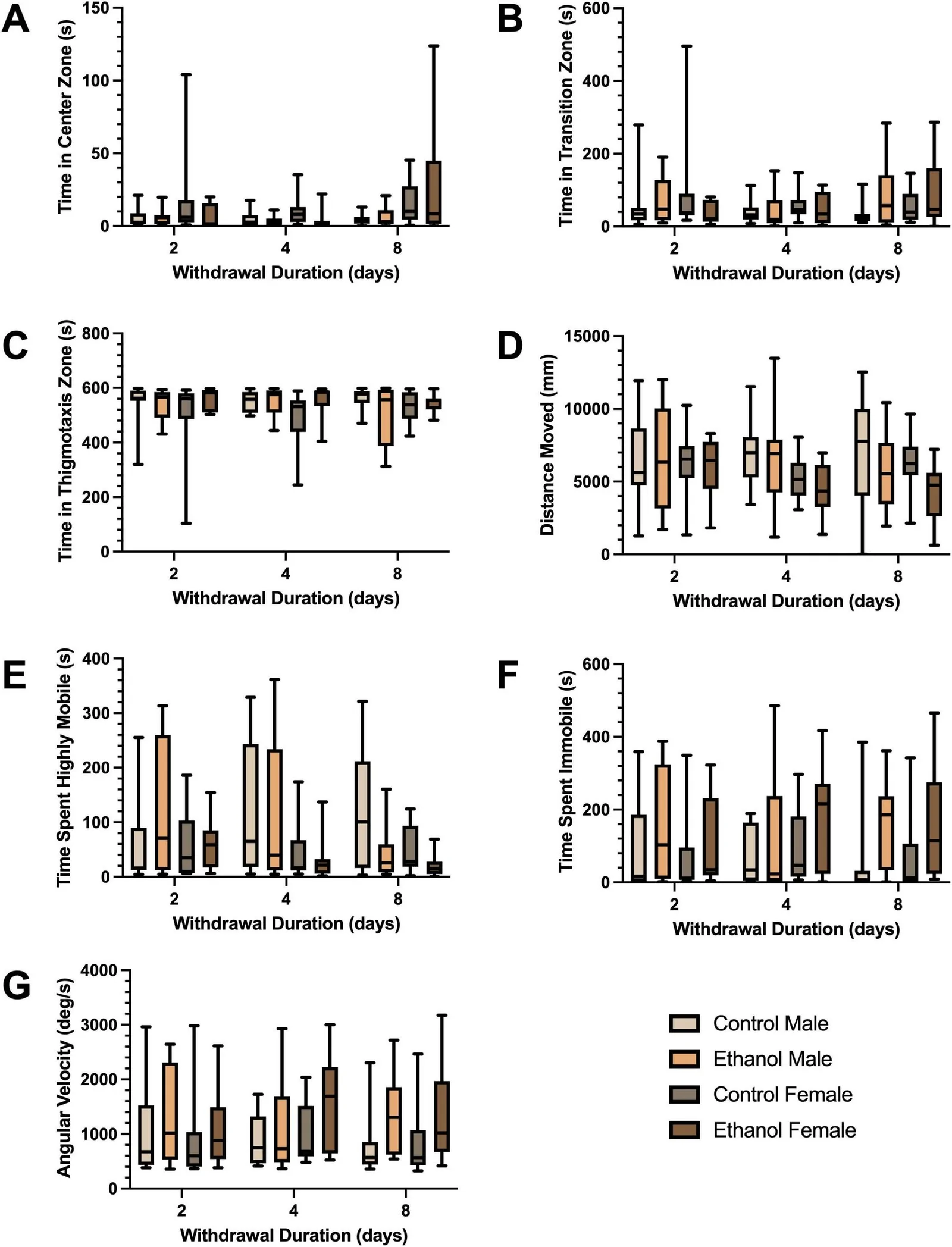
Sex differences in the open field test following condensed repeated ethanol dosing. Male and female zebrafish were assessed at 2, 4, and 8 days of withdrawal following repeated dosing. **(A)** Time spent in the center zone of the arena. Control females spent significantly more time in the center zone compared to control males **(B)** Time spent in the transition zone of the arena. **(C)** Time spent in the thigmotaxis zone of the arena. **(D)** Total distance moved during the trial. **(E)** Time spent highly mobile. **(F)** Time spent immobile. **(G)** Mean absolute angular velocity. Each box represents the 25^th^ and 75^th^ percentiles, with whiskers showing the smallest and largest values. The median is represented by the line within each box.

A three-way mixed-effects analysis revealed no significant three-way interaction for time spent in the center zone (*F*(2, 132) = 0.89, *p* = 0.415; Figure 3A). Likewise, none of the two-way interactions reached statistical significance: withdrawal duration × sex (*F*(2, 132) = 1.93, *p* = 0.149), withdrawal duration × treatment (*F*(2, 132) = 1.52, *p* = 0.222), or sex × treatment (*F*(1, 132) = 0.89, *p* = 0.633). A significant main effect of sex was revealed (*F*(1, 132) = 8.67, *p* = 0.004), with no significant effects of withdrawal duration (*F*(2, 132) = 2.57, *p* = 0.081) or treatment (*F*(1, 132) = 0.21, *p* = 0.645). A Holm-Šídák *post hoc* test revealed no significant differences between sex at any withdrawal duration.

#### Time in the transition zone

A three-way mixed-effects analysis revealed no significant three-way interaction for time spent in the transition zone (*F*(2, 86) = 0.78, *p* = 0.460; Figure 3B). The withdrawal duration × treatment interaction was significant (*F*(2, 86) = 3.49, *p* = 0.035); however, neither the withdrawal duration × sex interaction (*F*(2, 86) = 0.25, *p* = 0.782) nor the sex × treatment interaction (*F*(1, 46) = 1.31, *p* = 0.259) were significant. There were no significant main effects for withdrawal duration (*F*(2, 86) = 1.39, *p* = 0.254), sex (*F*(1, 46) = 0.32, *p* = 0.574), or treatment (*F*(1, 46) = 0.32, *p* = 0.577). A Holm-Šídák *post hoc* test revealed no significant differences between withdrawal duration and treatment groups at any withdrawal duration.

#### Time in the thigmotaxis zone

A three-way mixed-effects analysis revealed no significant three-way interaction for time spent in the thigmotaxis zone (*F*(2, 86) = 0.03, *p* = 0.973; Figure 3C). The sex × treatment interaction was significant (*F*(1, 46) = 6.02, *p* = 0.018); however, neither the withdrawal duration × sex interaction (*F*(2, 86) = 0.70, *p* = 0.499) nor the withdrawal duration × treatment interaction (*F*(2, 86) = 2.16, *p* = 0.122) were significant. There were no significant main effects for withdrawal duration (*F*(2, 86) = 0.01, *p* = 0.997), sex (*F*(1, 46) = 0.43, *p* = 0.516), or treatment (*F*(1, 46) = 0.62, *p* = 0.437). A Holm-Šídák *post hoc* test revealed no significant differences between sex and treatment groups at any withdrawal duration.

#### Distance moved

A three-way mixed-effects analysis revealed no significant three-way interaction for distance moved (*F*(2, 132) = 0.01, *p* = 0.994; Figure 3D). None of the two-way interactions reached statistical significance: withdrawal duration × sex (*F*(2, 132) = 1.01, *p* = 0.367), withdrawal duration × treatment (*F*(2, 132) = 1.18, *p* = 0.312), or sex × treatment (*F*(1, 132) = 0.64, *p* = 0.427). A significant main effect of sex was revealed (*F*(1, 132) = 5.94, *p* = 0.016), but no significant effects of withdrawal duration (*F*(2, 132) = 0.51, *p* = 0.599) or treatment *(F*(1, 132) = 3.60, *p* = 0.060) were found. A Holm-Šídák *post hoc* test revealed no significant differences between sex at any withdrawal duration.

#### Time spent highly mobile

A three-way mixed-effects analysis revealed no significant three-way interaction for time spent highly mobile (*F*(2, 84) = 1.37, *p* = 0.260; Figure 3E). The withdrawal duration × treatment interaction was significant (*F*(2, 84) = 3.47, *p* = 0.036); however, neither the withdrawal duration × sex interaction (*F*(2, 84 = 0.73, *p* = 0.486) nor the sex × treatment interaction (*F*(1, 46) = 0.19, *p* = 0.669) were significant. A significant main effect of sex was found (*F*(1, 46) = 9.61, *p* = 0.003), but no significant effects of withdrawal duration (*F*(2, 84) = 0.71, *p* = 0.494) or treatment (*F*(1, 46) = 0.36, *p* = 0.553) were found. A Holm-Šídák *post hoc* test revealed no significant differences between sex and treatment groups at any withdrawal duration.

#### Time spent immobile

A three-way mixed-effects analysis revealed no significant three-way interaction for time spent immobile (*F*(2, 132) = 0.30, *p* = 0.741; Figure 3F). None of the two-way interactions reached significance: withdrawal duration × sex (*F*(2, 132) = 0.94, *p* = 0.391), withdrawal duration × treatment (*F*(2, 132) = 0.47, *p* = 0.627), or sex × treatment (*F*(1, 132) = 0.02, *p* = 0.895). A significant main effect of treatment was revealed (*F*(1, 132) = 9.05, *p* = 0.003), but no significant effects of withdrawal duration (*F*(2, 132) = 0.02, *p* = 0.979), or sex (*F*(1, 132) = 0.12, *p* = 0.734) were found. A Holm-Šídák *post hoc* test revealed no significant differences between treatment groups at any withdrawal duration.

#### Mean absolute angular velocity

A three-way mixed-effects analysis revealed no significant three-way interaction for mean absolute angular velocity (*F*(2, 132) = 0.33, *p* = 0.720; Figure 3G). None of the two-way interactions reached significance: withdrawal duration × sex (*F*(2, 132) = 1.06, *p* = 0.350), withdrawal duration × treatment (*F*(2, 132) = 0.61, *p* = 0.544), or sex × treatment (*F*(1, 132) = 0.15, *p* = 0.700). A significant main effect of treatment was revealed (*F*(1, 132) = 8.54, *p* = 0.004), but no significant effects of withdrawal duration (*F*(2, 132) = 0.15, *p* = 0.863) or sex (*F*(1, 132) = 0.05, *p* = 0.823) were found. A Holm-Šídák *post hoc* test revealed no significant differences between treatment groups at any withdrawal duration.

## Discussion

This study used a condensed repeated ethanol dosing paradigm to investigate withdrawal-related anxiety-like behavior in adult zebrafish. Adult zebrafish were immersed in 0.8% ethanol three times daily for 5 consecutive days and assessed in an OF test at 2, 4, and 8 days of withdrawal. We found significant increases in immobility and angular velocity between ethanol-treated and control fish (main effect of treatment with no withdrawal time interaction). Furthermore, at 8 days of withdrawal, ethanol-treated fish showed significantly less time spent highly mobile, with increased time spent in the transition zone and significantly decreased time spent in the thigmotaxis zone compared to controls. Ethanol-treated fish did not differ significantly from controls in center zone preference at any duration of withdrawal. Together, these findings show that the condensed dosing paradigm produced measurable behavioral effects, but the resulting profile differed from the anxiety-like phenotype typically characterized by increased thigmotaxis preference (Blaser et al., 2010; Borba et al., 2022).

Increased freezing or immobility is often used as a proxy for anxiety-like behavior in adult zebrafish in open-field swimming assays (Blaser et al., 2010; Borba et al., 2022; Cachat et al., 2010; Baiamonte et al., 2016); however, others have found no drug-induced changes in locomotion alongside changes in anxiety-like behavior (Johnson and Hamilton, 2017). Immobility could also be an indicator of fear, which can be separated from anxiety-like behavior with pharmacological compounds like beta-carboline (Scatterty and Hamilton, 2024). Alongside the significant main effect of treatment on immobility, we also observed significantly reduced high mobility at 8 days of withdrawal in ethanol-treated fish. In addition, high mobility was significantly lower at 8 days than at 2 days of withdrawal in ethanol-treated fish, indicating that this initially negligible locomotor change developed progressively over the withdrawal period. Despite these findings, no significant differences were found in overall distance moved during the test, suggesting that ethanol withdrawal primarily altered locomotion patterns rather than general activity.

Altered locomotion, including rapid orientation changes measured by angular velocity, has been found to correlate with anxiety-like states, alongside spatial preference measures such as thigmotaxis and bottom-dwelling (Cachat et al., 2010; Tran and Gerlai, 2013; Tran et al., 2015). A significant main effect of treatment was observed for mean absolute angular velocity. This effect suggests that ethanol increases erratic swim patterns, and aligns with previous research on ethanol withdrawal, which has reported increased hyperactivity and erratic swimming associated with anxiety-like behavior in zebrafish (Cachat et al., 2010; Tran and Gerlai, 2013; Mathur and Guo, 2011).

A significant increase in time spent in the transition zone and decrease in time spent in the thigmotaxis zone in ethanol-treated fish at 8 days of withdrawal in the current study may suggest that the condensed dosing paradigm did not produce a traditional anxiogenic withdrawal response. Increased thigmotaxis preference is a robust metric of anxiety-like behaviors during an OF test (Blaser et al., 2010; Borba et al., 2022). Here, we observe the opposite, which may indicate that ethanol withdrawal did not elicit the expected anxiogenic spatial preference behaviors. It is possible that the observed preference could be due to a disinhibited behavioral state resulting from withdrawal effects. The incongruity between locomotor and spatial measures of anxiety-like behavior may reflect a differential effect of ethanol withdrawal on the behavioral measures studied. Secondly, the overriding responses may be locomotor alterations that are stronger than anxiogenic location preferences. In other words, the anxiogenic locomotor patterns may be independent to the location preference of the fish (and they happen be immobile and freeze outside of the thigmotaxis zone). Taken together, the observed increase in absolute angular velocity, decrease in high mobility, and increased time spent immobile later during withdrawal reflect alterations in arousal, locomotion, fear, and/or exploratory behavior due to ethanol withdrawal (Baiamonte et al., 2016; da Silva Chaves et al., 2018).

The significant interaction between withdrawal time and ethanol effect for high mobility with an effect only at day 8 of withdrawal suggests that the condensed exposure schedule may also produce a delayed behavioral withdrawal phenotype. This delay is consistent with previous studies showing that repeated alcohol exposure can produce lasting behavioral and neurobiological adaptations, which evolve over the course of withdrawal and depend on ethanol dose and duration (Gerlai et al., 2009; Tran and Gerlai, 2013; Becker and Mulholland, 2014; Koob and Le Moal, 2006; Hagen et al., 2025). Previous studies indicate that the exposure paradigm is a critical determinant of behavioral outcome (Holcombe et al., 2013; Gerlai et al., 2009; Krook et al., 2019). Repeated daily ethanol exposure in zebrafish can produce anxiogenic withdrawal-related behavioral changes, such as increased bottom-dwelling and reversed scototaxis (Holcombe et al., 2013; Tran et al., 2015). However, the direction and magnitude of these effects vary with dose, duration, and withdrawal period. For example, Holcombe et al. (2013) found that a daily moderate 21-day ethanol exposure schedule reversed the scototaxis preference relative to controls at 2 days of withdrawal, whereas a weekly binge schedule had no effect at that time. By day 9 of withdrawal, neither group showed a reversal of scototaxis preference. Alternatively, Tran and Gerlai (2013) employed a chronic ethanol dosing schedule over 22 days with a progressively increasing dose to examine tolerance and withdrawal-related adaptation. In that model, chronically exposed fish showed attenuated responses to an acute ethanol challenge and exhibited more erratic movement after one day of withdrawal, consistent with withdrawal-related adaptation (Tran and Gerlai, 2013). Delayed withdrawal effects were observed by Mathur and Guo (2011), who used an intermittent dosing schedule of 1% ethanol for 20 min daily over 8 consecutive days and did not detect significant differences in the light/dark choice test or novel tank diving test until the 6th and 7th day of withdrawal. Similarly, delayed withdrawal-induced behavioral changes were observed in a repeated daily dose experiment with drug seeking behavior occurring at 8-days of withdrawal after 21-days of 0.8% ethanol (Hagen et al., 2025; Krook et al., 2019). Together, these studies support the idea that the ethanol withdrawal phenotypes seem to become more pronounced from 2 days onward.

In both zebrafish and mammals, ethanol positively modulates GABAergic signaling as a GABA_A_ receptor agonist, producing biphasic effects that depend on concentration and duration during acute exposure (Goodman and Wong, 2020; Müller et al., 2019). Low to intermediate concentrations of ethanol can dampen excitatory transmission and result in anxiolytic-like effects, whereas higher concentrations can produce depressant-like effects (Borba et al., 2025; Gerlai et al., 2009; Müller et al., 2019). Chronic ethanol exposure induces neuroadaptations in many neurotransmitter systems involved in behavioral regulation, leading to anxiogenic-like symptoms upon withdrawal due to dysregulation of the hypothalamic-pituitary-interrenal axis and increased excitatory GABAergic transmission (da Silva Chaves et al., 2018; Becker and Mulholland, 2014). From a neurochemical perspective, repeated ethanol exposure contributes to oxidative stress, which is harmful to both mitochondrial complexes and the central nervous system and may require time to normalize following cessation, leading to delayed behavioral withdrawal symptoms such as anxiety and fear (Agostini et al., 2018; Müller et al., 2019; Bonassoli et al., 2011).

Across all treatments and withdrawal durations, there were no significant differences between male and female behavior. Notably, there were no differential responses to ethanol, suggesting that the condensed dosing paradigm did not affect males and females differently. Previously, male AB zebrafish were found to be more sensitive to acute ethanol exposure than females in the OF test (Vossen et al., 2022). Conversely, a 7-day repeated ethanol dosing study found that female short-fin zebrafish displayed increased activity and exploration of the center zone during the OF test on day one of withdrawal compared to males (Resmim et al., 2025). Sex differences in chronic ethanol exposure also depend strongly on dosing duration, behavioral endpoint, and strain. For example, a 10-week chronic exposure study found that female WT fish displayed increased sensitivity to 1.0% (vol/vol) ethanol compared to male fish, particularly during the final 5 weeks of treatment, an effect that was not observed in the long-fin striped fish (Dlugos et al., 2011). Other studies report no pharmacologically induced sex differences with repeated LSD (Hagen et al., 2024), conditioned colour preference to ethanol (Hagen et al., 2025), or acute exposure to ethanol, beta-carboline, beta caryophyllene, and tetrahydrocannabinol during the novel tank dive test (Johnson et al., 2024). The lack of sex differences in the present study may be due to the condensed nature of the dosing paradigm, insufficient sample sizes within each subgroup (decreased statistical power), genetic strain of the fish, or a combination of these factors.

Several limitations should be considered when interpreting the findings in this study. We relied solely on behavioral measures and did not assess differences in candidate neurochemical systems like glutamate, GABA, serotonin, or dopamine, nor did we assess changes in the hypothalamic-pituitary-interrenal axis, brain alcohol levels, or stress hormones such as cortisol, which are commonly involved in zebrafish ethanol withdrawal and anxiety-like behavior (Cachat et al., 2010; Tran et al., 2015). Future studies should vary ethanol concentration, exposure duration, number of doses per day, withdrawal intervals, and consider a combination of behavioral tests to optimize the condensed paradigm.

The overall goal of the current study was to develop a condensed repeated daily dosing paradigm that more accurately represents the stress-craving cycle seen in frequent drinkers at risk for AUD. The significant increases in immobility and mean absolute angular velocity and decreased high mobility at 8 days of withdrawal indicate a response phenotype to be further analyzed in future research. Furthermore, this condensed model is more efficient for high-throughput studies and may be useful with other pharmacological agents and AUD withdrawal treatments.

## Data Availability

The original contributions presented in this study are included in this article/Supplementary material, further inquiries can be directed to the corresponding author.

## References

[B1] Agostini J. F. Toé H. C. Z. D. Vieira K. M. Baldin S. L. Costa N. L. F. Cruz C. U. et al. (2018). Cholinergic system and oxidative stress changes in the brain of a zebrafish model chronically exposed to ethanol. *Neurotoxicity Res.* 33 749–758. 10.1007/s12640-017-9816-8 28942534

[B2] American Psychiatric Association (2022). *Diagnostic and Statistical Manual of Mental Disorders*, 5th Edn. Washington, DC: American Psychiatric Association.

[B3] Baiamonte M. Parker M. O. Vinson G. P. Brennan C. H. (2016). Sustained effects of developmental exposure to ethanol on zebrafish anxiety-like behaviour. *PLoS One* 11:e0148425. 10.1371/journal.pone.0148425 26862749 PMC4749633

[B4] Bauer B. Mally A. Liedtke D. (2021). Zebrafish embryos and larvae as alternative animal models for toxicity testing. *Intern. J. Mol. Sci.* 22:13417. 10.3390/ijms222413417 34948215 PMC8707050

[B5] Baumberg B. (2006). The global economic burden of alcohol: A review and some suggestions. *Drug Alcohol Rev.* 25 537–551. 10.1080/09595230600944479 17132572

[B6] Becker H. C. Lopez M. F. (2004). Increased ethanol drinking after repeated chronic ethanol exposure and withdrawal experience in C57BL/6 mice. *Alcohol. Clin. Exp. Res.* 28 1829–1838. 10.1097/01.ALC.0000149977.95306.3A 15608599

[B7] Becker H. C. Mulholland P. J. (2014). Neurochemical mechanisms of alcohol withdrawal. *Handb. Clin. Neurol.* 125 133–156. 10.1016/B978-0-444-62619-6.00009-4 25307573 PMC6943828

[B8] Blaser R. E. Chadwick L. McGinnis G. C. (2010). Behavioral measures of anxiety in zebrafish (Danio rerio). *Behav. Brain Res.* 208 56–62. 10.1016/j.bbr.2009.11.009 19896505

[B9] Bonassoli V. T. Milani H. De Oliveira R. M. W. (2011). Ethanol withdrawal activates nitric oxide–producing neurons in anxiety-related brain areas. *Alcohol* 45 641–652. 10.1016/j.alcohol.2010.11.007 21194876

[B10] Borba J. V. Biasuz E. Sabadin G. R. Savicki A. C. Canzian J. Luchiari A. C. et al. (2022). Influence of acute and unpredictable chronic stress on spatio-temporal dynamics of exploratory activity in zebrafish with emphasis on homebase-related behaviors. *Behav. Brain Res.* 435:114034. 10.1016/j.bbr.2022.114034 35914633

[B11] Borba J. V. Resmim C. M. Fontana B. D. Moraes H. S. Müller M. L. Blanco L. et al. (2025). Anxiogenic and anxiolytic modulators differentially affect thigmotaxis and thrashing behavior in adult zebrafish during habituation to the open field test. *Behav. Process.* 228:105199. 10.1016/j.beproc.2025.105199 40246212

[B12] Cachat J. Canavello P. Elegante M. Bartels B. Hart P. Bergner C. et al. (2010). Modeling withdrawal syndrome in zebrafish. *Behav. Brain Res.* 208 371–376. 10.1016/j.bbr.2009.12.004 20006651

[B13] Carvalho A. F. Heilig M. Perez A. Probst C. Rehm J. (2019). Alcohol use disorders. *Lancet* 394 781–792. 10.1016/S0140-6736(19)31775-1 31478502

[B14] Chapman R. Plaat F. (2009). Alcohol and anaesthesia. *Continuing Educ. Anaesthesia Crit. Care Pain* 9 10–13. 10.1093/bjaceaccp/mkn045

[B15] Clayman C. L. Malloy E. J. Kearns D. N. Connaughton V. P. (2017). Differential behavioral effects of ethanol pre-exposure in male and female zebrafish (Danio rerio). *Behav. Brain Res.* 335 174–184. 10.1016/j.bbr.2017.08.007 28797598

[B16] da Silva Chaves S. N. Felício G. R. Costa B. P. D. de Oliveira W. E. A. Lima-Maximino M. G. Siqueira Silva D. H. et al. (2018). Behavioral and biochemical effects of ethanol withdrawal in zebrafish. *Pharmacol. Biochem. Behav.* 169 48–58. 10.1016/j.pbb.2018.04.006 29689295

[B17] Dewari P. S. Ajani F. Kushawah G. Kumar D. S. Mishra R. K. (2016). Reversible loss of reproductive fitness in zebrafish on chronic alcohol exposure. *Alcohol* 50 83–89. 10.1016/j.alcohol.2015.11.006 26781213

[B18] Dlugos C. A. Rabin R. A. (2003). Ethanol effects on three strains of zebrafish: Model system for genetic investigations. *Pharmacol. Biochem. Behav.* 74 471–480. 10.1016/S0091-3057(02)01026-2 12479969

[B19] Dlugos C. A. Brown S. J. Rabin R. A. (2011). Gender differences in ethanol-induced behavioral sensitivity in zebrafish. *Alcohol* 45 11–18. 10.1016/j.alcohol.2010.08.018 20880661

[B20] Fachin-Scheit D. J. Frozino Ribeiro A. Pigatto G. Oliveira Goeldner F. Boerngen de Lacerda R. (2006). Development of a mouse model of ethanol addiction: Naltrexone efficacy in reducing consumption but not craving. *J. Neural Trans.* 113 1305–1321. 10.1007/s00702-005-0416-z 16465467

[B21] Fukushiro D. F. Josino F. S. Saito L. P. Berro L. F. Morgado F. Frussa-Filho R. (2012). Acute and chronic ethanol differentially modify the emotional significance of a novel environment: Implications for addiction. *Intern. J. Neuropsychopharmacol.* 15 1109–1120. 10.1017/S1461145711001283 21854680

[B22] Gerlai R. Chatterjee D. Pereira T. Sawashima T. Krishnannair R. (2009). Acute and chronic alcohol dose: Population differences in behavior and neurochemistry of zebrafish. *Genes Brain Behav.* 8 586–599. 10.1111/j.1601-183X.2009.00488.x 19243447 PMC2880629

[B23] Godwin J. Sawyer S. Perrin F. Oxendine S. E. Kezios Z. D. (2012). “Adapting the Open Field Test to Assess Anxiety-Related Behavior in Zebrafish,” in *Zebrafish Protocols for Neurobehavioral Research*, eds Kalueff A. V. Stewart A. M. (Totowa, NJ: Humana Press), 181–189. 10.1007/978-1-61779-597-8_13

[B24] Goodman A. C. Wong R. Y. (2020). Differential effects of ethanol on behavior and GABAA receptor expression in adult zebrafish (Danio rerio) with alternative stress coping styles. *Sci. Rep.* 10:13076. 10.1038/s41598-020-69980-2 32753576 PMC7403336

[B25] Hagen E. V. Schalomon M. Zhang Y. Hamilton T. J. (2024). Repeated microdoses of LSD do not alter anxiety or boldness in zebrafish. *Sci. Rep.* 14:4389. 10.1038/s41598-024-54676-8 38388550 PMC10883969

[B26] Hagen E. V. Zhang Y. Hamilton T. J. (2025). From colours to cravings: Exploring conditioned colour preference to ethanol in zebrafish. *Pharmacol. Biochem. Behav.* 246:173909. 10.1016/j.pbb.2024.173909 39579875

[B27] Hendriks H. F. J. (2020). Alcohol and human health: What is the evidence? *Ann. Rev. Food Sci. Technol.* 11 1–21. 10.1146/annurev-food-032519-051827 32209032

[B28] Holcombe A. Howorko A. Powell R. A. Schalomon M. Hamilton T. J. (2013). Reversed scototaxis during withdrawal after daily-moderate, but not weekly-binge. Administration of Ethanol in Zebrafish. *PLoS One* 8:e63319. 10.1371/journal.pone.0063319 23675478 PMC3652870

[B29] Howe K. Clark M. D. Torroja C. F. Torrance J. Berthelot C. Muffato M. et al. (2013). The zebrafish reference genome sequence and its relationship to the human genome. *Nature* 496 498–503. 10.1038/nature12111 23594743 PMC3703927

[B30] Hughes S. Hessel E. V. S. (2024). Zebrafish and nematodes as whole organism models to measure developmental neurotoxicity. *Crit. Rev. Toxicol.* 54 330–343. 10.1080/10408444.2024.2342448 38832580

[B31] Jesse S. Bråthen G. Ferrara M. Keindl M. Ben-Menachem E. Tanasescu R. et al. (2016). Alcohol withdrawal syndrome: Mechanisms, manifestations, and management. *Acta Neurol. Scand.* 135:4. 10.1111/ane.12671 27586815 PMC6084325

[B32] Johnson A. L. Hurd P. L. Hamilton T. J. (2024). Sex, drugs, and zebrafish: Acute exposure to anxiety-modulating compounds in a modified novel tank dive test. *Pharmacol. Biochem. Behav.* 243:173841. 10.1016/j.pbb.2024.173841 39074564

[B33] Johnson A. L. Hurd P. L. Mathot K. J. Hamilton T. J. (2025). Behavioural variability and repeatability in adult zebrafish (Danio rerio) using the novel tank dive test. *PLoS One* 20:e0335308. 10.1371/journal.pone.0335308 41144504 PMC12558523

[B34] Johnson A. Hamilton T. J. (2017). Modafinil decreases anxiety-like behaviour in zebrafish. *PeerJ* 5:e2994. 10.7717/peerj.2994 28229024 PMC5312568

[B35] Johnson A. Loh E. Verbitsky R. Slessor J. Franczak B. C. Schalomon M. et al. (2023). Examining behavioural test sensitivity and locomotor proxies of anxiety-like behaviour in zebrafish. *Sci. Rep.* 13:3768. 10.1038/s41598-023-29668-9 36882472 PMC9992706

[B36] Kitson J. E. Ord J. Watt P. J. (2022). Maternal chronic ethanol exposure decreases stress responses in zebrafish offspring. *Biomolecules* 12:1143. 10.3390/biom12081143 36009037 PMC9405564

[B37] Knapp D. J. Breese G. R. (2012). “Models of Chronic Alcohol Exposure and Dependence,” in *Psychiatric Disorders: Methods and Protocols*, ed. Kobeissy F. H. (Totowa, NJ: Humana Press), 205–230. 10.1007/978-1-61779-458-2_13 22231816

[B38] Koob G. F. Le Moal M. (2006). “CHAPTER 5—Alcohol,” in *Neurobiology of Addiction* (Elsevier), 173–241. 10.1016/B978-012419239-3/50042-4

[B39] Krook J. T. Duperreault E. Newton D. Ross M. S. Hamilton T. J. (2019). Repeated ethanol exposure increases anxiety-like behaviour in zebrafish during withdrawal. *PeerJ* 7:e6551. 10.7717/peerj.6551 30842911 PMC6397752

[B40] Mathur P. Guo S. (2011). Differences of acute versus chronic ethanol exposure on anxiety-like behavioral responses in zebrafish. *Behav. Brain Res.* 219 234–239. 10.1016/j.bbr.2011.01.019 21255611 PMC3062742

[B41] McGovern P. E. Zhang J. Tang J. Zhang Z. Hall G. R. Moreau R. A. et al. (2004). Fermented beverages of pre- and proto-historic China. *Proc. Natl. Acad. Sci. U. S. A.* 101 17593–17598. 10.1073/pnas.0407921102 15590771 PMC539767

[B42] Müller T. E. Nunes M. E. M. Rodrigues N. R. Fontana B. D. Hartmann D. D. Franco J. L. et al. (2019). Neurochemical mechanisms underlying acute and chronic ethanol-mediated responses in zebrafish: The role of mitochondrial bioenergetics. *Neurochem. Intern.* 131:104584. 10.1016/j.neuint.2019.104584 31654679

[B43] Müller T. E. Nunes S. Z. Silveira A. Loro V. L. Rosemberg D. B. (2017). Repeated ethanol exposure alters social behavior and oxidative stress parameters of zebrafish. *Prog. Neuro-Psychopharmacol. Biol. Psychiatry* 79 105–111. 10.1016/j.pnpbp.2017.05.026 28602852

[B44] Nainu F. Jota Baptista C. Faustino-Rocha A. Oliveira P. A. (2023). Editorial: Model organisms in experimental pharmacology and drug discovery 2022. *Front. Pharmacol.* 14:1143934. 10.3389/fphar.2023.1143934 37063289 PMC10096023

[B45] NIAAA (2001). *Craving Research: Implications for Treatment.* Bethesda, MD: NIAAA.

[B46] Nowicki M. Tran S. Chatterjee D. Gerlai R. (2015). Inhibition of phosphorylated tyrosine hydroxylase attenuates ethanol-induced hyperactivity in adult zebrafish (Danio rerio). *Pharmacol. Biochem. Behav.* 138 32–39. 10.1016/j.pbb.2015.09.008 26366782 PMC4625845

[B47] Pittman J. T. Ichikawa K. M. (2013). iPhone® applications as versatile video tracking tools to analyze behavior in zebrafish (Danio rerio). *Pharmacol. Biochem. Behav.* 106 137–142. 10.1016/j.pbb.2013.03.013 23558086

[B48] Pittman J. Hylton A. (2015). Behavioral, endocrine, and neuronal alterations in zebrafish (Danio rerio) following sub-chronic coadministration of fluoxetine and ketamine. *Pharmacol. Biochem. Behav.* 139 158–162. 10.1016/j.pbb.2015.08.014 26303305

[B49] Resmim C. M. Borba J. V. Gonçalves F. L. Santos L. W. Canzian J. Fontana B. D. et al. (2025). Understanding sex and populational differences in spatio-temporal exploration patterns and homebase dynamics of zebrafish following repeated ethanol exposure. *Prog. Neuro-Psychopharmacol. Biol. Psychiatry* 136:111171. 10.1016/j.pnpbp.2024.111171 39395733

[B50] Scatterty K. R. Hamilton T. J. (2024). β-Carboline (FG-7142) modulates fear but not anxiety-like behaviour in zebrafish. *Sci. Rep.* 14:668. 10.1038/s41598-023-51072-6 38182703 PMC10770314

[B51] Spence R. Gerlach G. Lawrence C. Smith C. (2008). The behaviour and ecology of the zebrafish. Danio rerio. *Biol. Rev.* 83 13–34. 10.1111/j.1469-185X.2007.00030.x 18093234

[B52] Stahre M. (2014). Contribution of excessive alcohol consumption to deaths and years of potential life lost in the United States. *Prevent. Chronic Dis.* 11:E109. 10.5888/pcd11.130293 24967831 PMC4075492

[B53] Stewart A. M. Gaikwad S. Kyzar E. Kalueff A. V. (2012). Understanding spatio-temporal strategies of adult zebrafish exploration in the open field test. *Brain Res.* 1451 44–52. 10.1016/j.brainres.2012.02.064 22459042

[B54] Swift R. M. Aston E. R. (2015). Pharmacotherapy for alcohol use disorder: Current and emerging therapies. *Harvard Rev. Psychiatry* 23 122–133. 10.1097/HRP.0000000000000079 25747925 PMC4790835

[B55] Tran S. Gerlai R. (2013). Time-course of behavioural changes induced by ethanol in zebrafish (Danio rerio). *Behav. Brain Res.* 252 204–213. 10.1016/j.bbr.2013.05.065 23756142 PMC3804555

[B56] Tran S. Chatterjee D. Gerlai R. (2015). An integrative analysis of ethanol tolerance and withdrawal in zebrafish (Danio rerio). *Behav. Brain Res.* 276 161–170. 10.1016/j.bbr.2014.02.034 24598276 PMC4225192

[B57] Vengeliene V. Bilbao A. Molander A. Spanagel R. (2008). Neuropharmacology of alcohol addiction. *Br. J. Pharmacol.* 154 299–315. 10.1038/bjp.2008.30 18311194 PMC2442440

[B58] Vossen L. E. Brunberg R. Rådén P. Winberg S. Roman E. (2022). Sex-Specific effects of acute ethanol exposure on locomotory activity and exploratory behavior in adult zebrafish (Danio rerio). *Front. Pharmacol.* 13:853936. 10.3389/fphar.2022.853936 35721152 PMC9201571

[B59] Wills T. A. Knapp D. J. Overstreet D. H. Breese G. R. (2009). Sensitization, duration, and pharmacological blockade of anxiety-like behavior following repeated ethanol withdrawal in adolescent and adult rats. *Alcohol. Clin. Exp. Res.* 33 455–463. 10.1111/j.1530-0277.2008.00856.x PMC2847263 19120055

[B60] Witkiewitz K. Litten R. Z. Leggio L. (2019). Advances in the science and treatment of alcohol use disorder. *Sci. Adv.* 5:eaax4043. 10.1126/sciadv.aax4043 31579824 PMC6760932

[B61] Wood E. Bright J. Hsu K. Goel N. Ross J. W. G. Hanson A. et al. (2023). Canadian guideline for the clinical management of high-risk drinking and alcohol use disorder. *Can. Med. Assoc. J.* E1364–E1379. 10.1503/cmaj.230715 37844924 PMC10581718

